# Gender differences in patients attending Early Psychosis Intervention Programme

**DOI:** 10.1192/j.eurpsy.2022.1991

**Published:** 2022-09-01

**Authors:** A.B. Mulero Garcia, E. Sánchez Martínez, C. Gómez Sanchez-Lafuente

**Affiliations:** Hospital Regional Universitario de Málaga, Ugc Salud Mental, Málaga, Spain

**Keywords:** Gender differences, Early Psychosis Intervention Programme, Psychosis, female gender

## Abstract

**Introduction:**

Studing the scope of differences found in terms of gender in First Psychotic Episodes patients, should enhance our understanding of such disorders and improve the therapeutic approaches.

**Objectives:**

Our main objective was to compare the sociodemographic variables between men and women included in the Early Intervention Program in Psychosis of the Regional University Hospital of Malaga between the years 2016-2020.

**Methods:**

Retrospective study in which the characteristics of 135 patients who started the Early Intervention Program in Psychosis of the Regional University Hospital of Malaga between the years 2016-2020 were analyzed. Statistical analysis was performed using SPSS 25.0. For the comparison of variables, Student’s t was used for quantitative variables and Chi square for dichotomous variables.

**Results:**

Patients included in the Program; 32% were women and 68% were men. The average of age at the beginning was 35.56 for women and 28,47 for men. Most of the women were married and most of the men were single. The majority of men lived with their original family and for women with their own family. In relation with the consumption of toxins, alcohol and to smoke, were more frequent in men. More results in poster.

**Conclusions:**

Despite the limitations due to our study design, the results obtained are in agreement with some of the discussions that are currently in force. Given that, female gender is associated with lower risk of psychosis, better premorbid adjustment, lower drug consumption and a later onset of the disease in the first-episode psychosis.

**Disclosure:**

No significant relationships.

